# Grasping multiple sclerosis: do quantitative motor assessments provide a link between structure and function?

**DOI:** 10.1007/s00415-012-6639-7

**Published:** 2012-08-08

**Authors:** R. Reilmann, F. Holtbernd, R. Bachmann, S. Mohammadi, E. B. Ringelstein, M. Deppe

**Affiliations:** 1 Department of Neurology, University Clinic Muenster (UKM), Westfaelische-Wilhelms-University Muenster, Albert-Schweitzer Campus 1, Building A1, 48149 Muenster, Germany; 2 Department of Radiology, University Clinic of Muenster, Westfaelische-Wilhelms-University Muenster, Albert-Schweitzer Campus 1, 48149 Muenster, Germany; 3 Department of Radiology, Marienhospital Aachen, Zeise 4, 52066 Aachen, Germany

**Keywords:** Multiple sclerosis, DTI, Clinical neurophysiology, Outcome research

## Abstract

Motor disability in MS is commonly assessed by the Expanded Disability Status Scale (EDSS). Categorical rating scales are limited by subjective error and inter-rater variability. Therefore, objective and quantitative measures of motor disability may be useful to supplement the EDSS in the setting of clinical trials. It was previously shown that grip-force-variability (GFV) is increased in MS. We hypothesized that GFV may be an objective measure of motor disability in MS. To investigate whether the increase in GFV in MS is correlated to the clinical disability as assessed by the EDSS and to microstructural changes in the brain as assessed by diffusion tensor imaging, GFV was recorded in a grasping and lifting task in 27 MS patients and 23 controls using a grip-device equipped with a force transducer. The EDSS was assessed by neurologists experienced in MS. Patients underwent diffusion tensor imaging at 3T to assess the fractional anisotropy (FA) of the cerebral white matter as a measure of microstructural brain integrity. GFV was increased in MS and correlated to changes in the FA of white matter in the vicinity of the somatosensory and visual cortex. GFV also correlated with the EDSS. GFV may be a useful objective measure of motor dysfunction in MS linked to disability and structural changes in the brain. Our data suggests that GFV should be further explored as an objective measure of motor dysfunction in MS. It could supplement the EDSS, e.g., in proof of concept studies.

## Introduction

Multiple sclerosis (MS) is an autoimmune central nervous system disorder resulting in demyelination and subsequent neuroaxonal damage. Impairments in motor coordination and loss of sensory perception are common in all forms of MS [4]. In clinical settings, disability—including sensory-motor dysfunction—is usually assessed by the Expanded Disability Status Scale (EDSS) [26], which frequently serves as outcome measure in clinical trials [27]. However, the EDSS is a clinical rating scale confined by inter- and intra-rater variability and limited sensitivity due to its categorical nature [16]. In contrast, supplementary objective and quantitative measures of motor dysfunction may improve the sensitivity of motor phenotype assessment in clinical trials, e.g., by reducing cohorts required to sufficiently power proof of concept studies.

Manipulation of objects in the precision grip (between thumb and index finger) is a motor task with high functional relevance in everyday life. Assessment of grip forces during grasping and lifting paradigms was able to objectively and quantitatively detect deficits in subjects with MS [28]. This was confirmed independently [24, 25]. Increased variability of motor performance, expressed by the variability of grip forces during a static holding phase, was a finding reported across all of these studies [24, 25, 28]. However, it is unknown whether changes in grip force variability (GFV) are correlated to the severity of motor symptoms and linked to changes observed in the brains of MS subjects. Several MRI techniques have been established to assess disease burden in the brains of subjects with MS; diffusion tensor imaging (DTI) has evolved as a reliable method to detect and monitor microstructural brain tissue damage in MS (for review see [9]). DTI was previously shown to correlate to clinical disability in MS [34, 41, 44] and is considered a promising imaging endpoint for proof of concept studies [11]. A DTI measure of anisotropy frequently applied in neurology and particularly in MS is “fractional anisotropy” (FA) (e.g., [2, 5, 6, 9]), which was predefined as a primary DTI outcome measure in this study.

We therefore decided to investigate whether changes in GFV are correlated to the disability detected in the EDSS and to changes of FA in DTI as a measure of microstructural white matter integrity. We hypothesized that GFV is (1) increased in subjects with MS compared to healthy controls, (2) correlated to disease severity and clinical disability as assessed by the EDSS, and (3) correlated to changes in FA as assessed by DTI.

## Subjects and methods

### Subjects

Twenty-seven subjects with MS according to the revised McDonald Criteria [36] (15 relapsing–remitting—RRMS, 8 secondary-progressive—SPMS, 4 primary-progressive MS—PPMS), 9 males and 18 females, mean age 39.3 ± 10.3 (all values mean ± SD, range 24–61), and 23 healthy control subjects, 7 males and 16 females, mean age 38.4 ± 9.3 (range 24–55) participated in the study after giving their written informed consent in accordance with the Declaration of Helsinki. Median EDSS was 4 (range 1–6.5). Exclusion criteria were: coexisting neurological diseases, orthopaedic disorders, or other impairments interfering with task performance. Control subjects had no neurological or psychiatric diseases and neurological examination was normal.

### Experimental setup and motor task

**Fig. 1:**
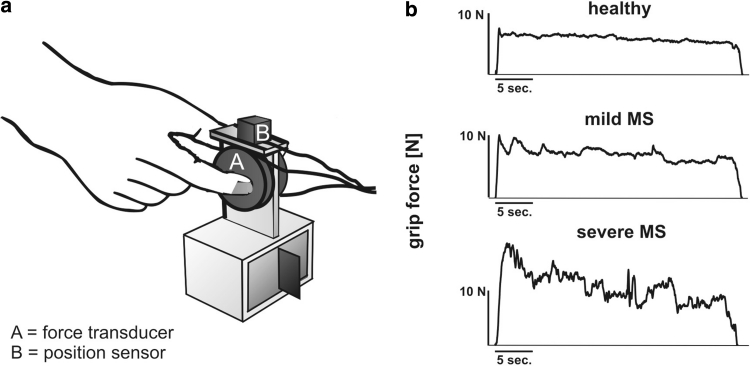
Quantitative motor assessment of multiple sclerosis using grip force variability method. **a** Grip device with force transducer and position sensor; **b** sample recordings of a control subject, a mild and severely affected patient with MS

Quantitative motor assessment was performed using a grip device (250 g) (see Fig. 1a) that was grasped and lifted in the precision grip between thumb and index finger as described before [37]. In brief, a pre-calibrated force transducer covered with 200-grit sandpaper (Nano 40, ATI, USA) measured grip and lift forces (0.025 N resolution) of the thumb. An electromagnetic 3D-sensor (Fastrack, Polhemus, USA) assessed the position of the device. Ten trials were performed after completion of three test trials. Once lifted, subjects held the object stable close to a marker located 10 cm above the table for 30 s.

The mean isometric grip force (GF[N]) and grip force variability (GFV, expressed as coefficient of variation = SD/GF × 100 [%]) were assessed during the static holding phase, which was pre-defined as the period from second 8 to 30 in each trial to exclude changes in the forces occurring during lift initiation (see Fig. 1b). Grip force assessments were performed in the morning to ensure that measurements were not affected by fatigue. The mean value of all ten trials was used for further analysis. Patients not able to perform all ten trials were excluded from the study. The task was performed using the right hand (dominant in 26 patients and 21 controls). Control subjects also performed the task using their left hand. Measures were not different between the dominant and nondominant hand (*p* = 0.15 for GFV; *p* = 0.53 for GF; paired *t* test). Therefore we decided to also include the two left-handed controls and the one left-handed MS subject to increase statistical power.

### DTI protocol

Twenty-three of the 27 MS patients underwent DTI. The remaining four did not tolerate the scan. MRI was performed using a 3T whole-body scanner (Gyroscan Intera T30, Philips, the Netherlands). Data were acquired using a single shot echo planar imaging (EPI) sequence in 72 axial slices (1.8 mm thick, no gap, FOV 230 × 230 mm, acquired matrix 127 × 128, *b* factors: 0 and 1,000 s/mm^2^ 6 gradient directions, 3 averages). For further processing all EPI images were reconstructed to 2.0 × 2.0 × 2.0 mm^3^. All images were spatially registered by the multicontrast image registration toolbox for optimal spatial pre-processing of DTI data prior to statistical analysis [29] and corrected for eddy currents in all three dimensions using a recently developed technique [6, 30]. After image registration all DTI images corresponded to the Montreal Neurological Institute (MNI) coordinate space.

In DTI microstructural brain tissue, alterations are frequently described by FA changes as the primary outcome measure [9]. However the FA is not the only parameter used for describing diffusion properties of brain tissue. FA is calculated from the three tensor eigenvalues λ_1_, λ_2_, and λ_3_ and is sensitive to differences between these tensor invariants: FA = sqrt((λ_1_ − λ_2_)^2^ + (λ_2_ − λ_3_)^2^ + (λ_1_ − λ_3_)^2^)/sqrt(2(λ_1_^2^ + λ_2_^2^ + λ_3_^2^)) [5]. The largest eigenvalue λ_1_ represents the apparent diffusion coefficient in direction of strongest (main) diffusion and is also denoted as axial diffusivity (AD). The eigenvalues λ_2_ and λ_3_ describe the diffusion perpendicular to the main diffusion direction and can be summarized as the radial diffusivity RD = (λ_2_ + λ_3_)/2. The average of all three eigenvalues is denominated as mean diffusivity MD = (λ_1_ + λ_2_ + λ_3_)/3 [1].

We calculated FA (predefined primary DTI outcome measure), AD, MD, and RD images of all patients and applied voxel-based statistics (VBS) using SPM (http://www.fil.ion.ucl.ac.uk/spm) to investigate the correlation between these four parameters and GFV on a voxel-by-voxel basis (4 mm FWHM, *p* < 0.01, corrected). In addition, we employed a region of interest (ROI)-based regression analysis to assess FA, AD, MD, and RD changes in relation to the patients’ GFV. The ROI was defined post hoc on the basis of the SPM results.

### Data processing and statistical analysis of behavioural data

Data was recorded (sampling rate of 400 Hz) and processed using a flexible data acquisition system (SC/ZOOM, Department of Physiology, University of Umea, Sweden). Statistical analysis was performed using SPSS14^®^. Student’s *t* test was calculated for intergroup comparisons between MS subjects and controls, the paired *t* test was used for intragroup comparisons; Pearson’s correlation coefficients were calculated to analyse correlations of GF and GFV with the EDSS. Statistical significance was assumed at *p* ≤ 0.05. Results were expressed in means ± standard-error-of-mean (SEM).

## Results

### Intergroup comparisons

**Fig. 2:**
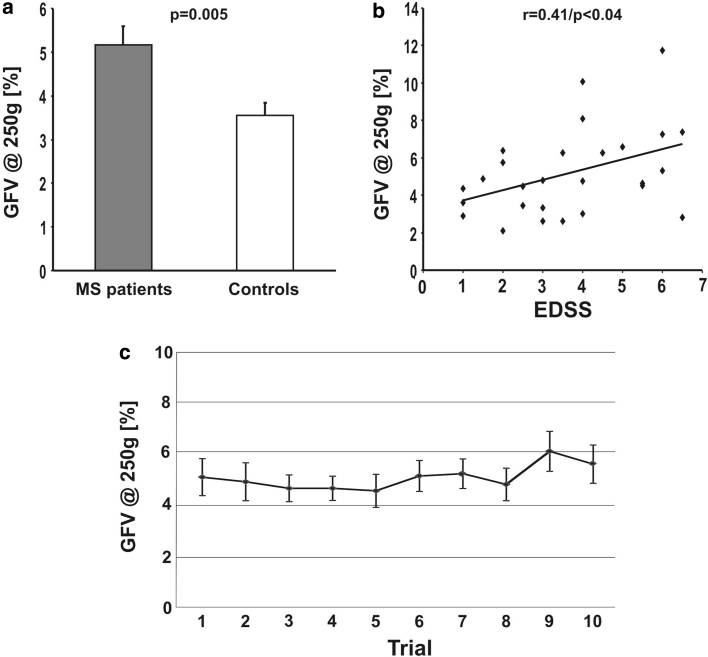
Grip force variability (GFV) in MS is increased compared to controls and correlated to the EDSS. **a** Increased GFV in MS patients compared to controls; **b** correlation of GFV and the EDSS; **c** intraclass correlation (ICC) of mean GFV measures across all ten trials in the MS group showed high agreement across trials (*r* = 0.89; *p* < 0.0001) indicating a high test–retest reliability of GFV (see text for details); *bars* indicate the standard error of mean

GFV was significantly increased in subjects with MS compared to controls (5.2 ± 0.4 % vs. 3.6 ± 0.3 %; *p* = 0.005) (see Fig. 2a). The mean applied GF did not differ significantly between groups (3.4 ± 0.3 N vs. 4.2 ± 0.4 N; *p* = 0.13). To determine the test–retest reliability of GFV, we calculated the intraclass correlation coefficient (ICC) of mean GFV measures across all ten trials in the MS group (see Fig. 2c). The ICC showed a robust agreement across trials (*r* = 0.89, *p* < 0.0001). We also calculated the ICC for the first and last five trials, respectively, to account for possible fatigue effects. We did not find significant differences between the two trial groups (*r* = 0.82 for the first five trials; *r* = 0.81 for the last five trials; *p* < 0.0001). Figure 2c visually implies that the last two trials might show an increased GFV compared to baseline. However, statistical analysis revealed no difference in GFV between the first and the two last trials (*p* = 0.56 for trial 1 vs. 10; *p* = 0.29 for trial 1 vs. 9; paired *t* test). We also investigated the relationship of grip force measures and age. Neither GF nor GFV were correlated with age in any of the groups (GF: *r* = 0.12, *p* = 0.55; GFV: *r* = 0.003, *p* = 0.98 for the MS group; GF: *r* = 0.29, *p* = 0.17; GFV = 0.29, *p* = 0.17 for the controls).

### Correlation of measures with the EDSS

GFV correlated significantly with the patients’ EDSS (*r* = 0.41; *p* < 0.04) (see Fig. 2b). Mean GF did not correlate with the EDSS (*r* = −0.2; *p* = 0.92).

### Correlation of GFV with FA, AD, RD, and MD

**Fig. 3:**
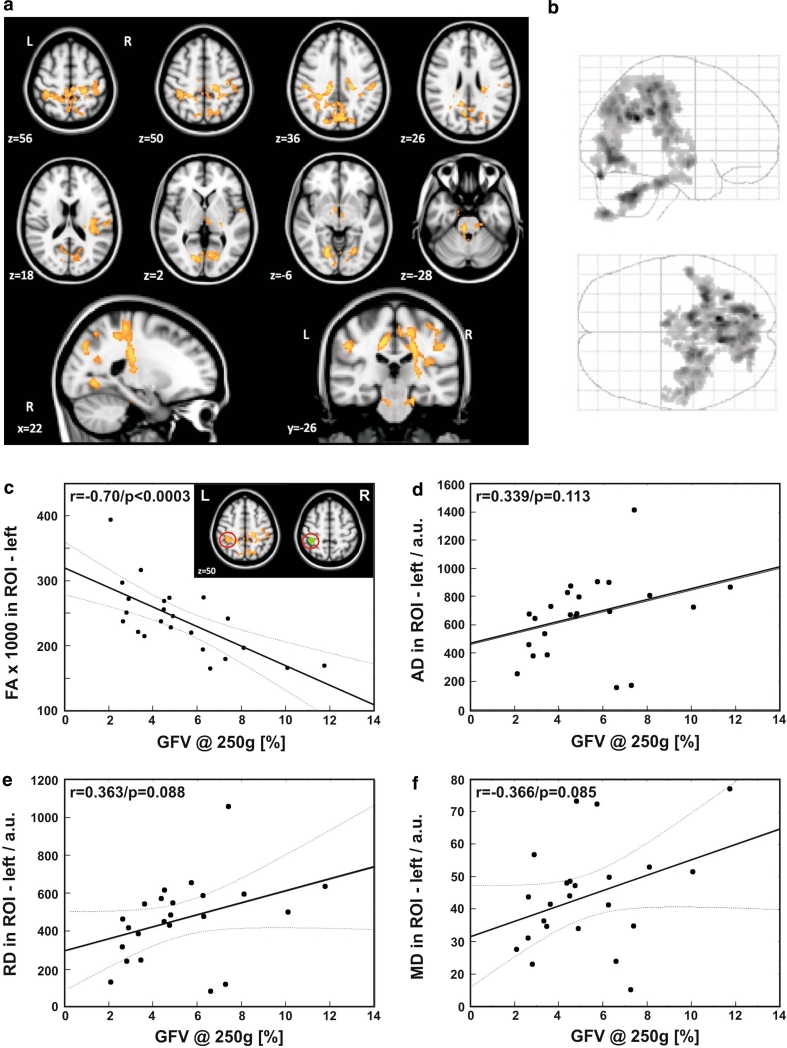
Link between structure and function: quantitative motor deficits are correlated to changes in FA. **a** Significant correlations between white matter FA reduction and GFV were found in areas associated with the primary somatosensory cortex and in the vicinity of the visual cortex (*p* < 0.0001, corrected for multiple comparisons, minimal cluster size 1,000 voxel, *orange rendered regions* represent *t* values between 1.72 and 6.11); **b** glass brain showing the areas of correlation; **c** regression analysis in ROI (post hoc) depicting high correlation between FA and GFV in the left hemisphere—see *green area* on right inlay—respective regions on both inlay images are marked by red circles; **d**–**f** regression analyses in the same ROI for AD, RD and MD are not significant (*p* > 0.05), but exhibit weak trends for all measures as may be appreciated in the plots. The biological meaning of these observations needs to be re-addressed in larger studies (*AD* axial diffusivity, *a.u.* arbitrary units, *FA* fractional anisotropy, *GFV* grip force variability, *MD* mean diffusivity, *RD* radial diffusivity)

The voxel-level SPM analysis revealed that GFV correlated significantly with regional FA of the white matter in several regions bilaterally (see Fig. 3a, b). Significant correlations between GFV and FA were found in the white matter regions associated to the primary somatosensory cortex. Additionally, we found strong correlations between GFV and FA of white matter in the vicinity of the visual cortex, whereas no correlations between GFV and FA could be observed in the white matter adjacent to the primary motor cortex or in the frontal white matter. Representative regression of GFV and FA across a ROI encompassing an area of high correlation in the left hemisphere (*r* = −0.70; *p* < 0.0003) is shown in Fig. 3c. Voxel-level analysis revealed no correlation between GFV and AD, RD, and MD after correction for multiple comparisons (*p* > 0.05). Thus, a ROI outlining prominent regions of significant correlations between GFV and AD, RD, and MD could not be defined. GFV did not significantly (*p* > 0.05) correlate with AD, RD, and MD in the ROI used for assessing quantitative FA changes (Fig. 3c), although trends for weak correlations were observed (*r*_AD_ = 0.34, *p*_AD_ = 0.11; *r*_RD_ = 0.37, *p*_RD_ < 0.09; *r*_MD_ = 0.36, *p*_MD_ < 0.09) as shown in Fig. 3d–f. Mean GF did not correlate with FA, AD, RD, and MD, neither at the whole brain level nor in the ROI analysis.

## Discussion

This study demonstrates that GFV is increased in subjects with MS. The amplitude of increase is correlated to the clinical disability as assessed by the EDSS and to microstructural changes in the brain measured by the FA of DTI of the central white matter. Furthermore, intraclass correlation analysis revealed a high test–retest reliability of the GFV assessment. We therefore confirmed our hypotheses and conclude that our results support a link between quantitative motor deficits, disability and structural changes in subjects with MS.

While increased GFV in MS was described previously in smaller cohorts [24, 25], this study is the first to demonstrate a link between GFV and changes in imaging and the EDSS. Assessment of DTI has been intensely studied in MS in order to provide an objective outcome measure of microstructural brain damage for clinical trials (e.g., [8, 11, 41, 44]). The direct link to brain pathology makes DTI particularly compelling for assessing novel measures such as GFV; a similar strategy has recently been applied to provide evidence for a link of oculomotor deficits to changes in DTI [12]. In another study, FA and individual radial diffusivities proved to be important markers of motor disabilities in MS patients, and FA exhibited a correlation with the EDSS [34]. Interestingly, in our study the correlation of the GFV with FA was stronger than with the EDSS. This may be explained by the quantitative and objective nature of both GFV and FA, while the EDSS is categorical and may be influenced by intra- and inter-rater variability. In addition, the EDSS does not specifically assess fine motor control of the hand. We also acknowledge that the EDSS is influenced by spinal pathology, which is not assessed by brain FA. Notably, although we observed a trend towards a weak correlation of GFV and individual diffusivity measures (i.e., AD, MD, and RD) in the ROI exhibiting highest correlation with FA, only FA significantly correlated with behavioural measures, suggesting that FA may be the most sensitive DTI measure to detect microstructural white matter damage associated with grip force control in our patient sample. However, statistically significant correlations of AD, MD, or RD with GFV might be observed in a larger cohort.

Appropriate coordination of upper extremities including grasping of objects is required for various tasks of daily living and impairments are linked to functional decline in MS [7]. The mechanisms governing grip force control in precision grasping are complex [20]. Permanent updating of afferent information is required to adjust motor output [21, 22]. Part of this afferent feedback is provided by mechanoreceptors in the skin of the digits [13, 43]. Lack of sensory information from the grasping fingers has been shown to cause a disturbance of grip force scaling [19, 32, 33]. In addition, vision is an important source of afferent information about the object’s characteristics and is used to adjust motor commands prior to the lift and during the grip [14, 17], affecting “feed-forward” mechanisms [31] and maintenance of stable grip forces during task performance [39]. The key importance of sensory and visual feedback mechanisms for grip force control suggests that both peripheral and central pathology in these neuronal pathways may disrupt force coordination [18]. Interestingly significant correlations between GFV and FA changes in our cohort were primarily localized to the white matter in the vicinity of the somatosensory and visual cortex, as shown in Fig. 3a, b. Afferent visual pathways are commonly affected in MS [23, 35] and changes in the white matter of the occipital, parietal and temporal lobes are seen across different forms of MS [3, 42]. Notably, neither GFV nor the mean applied grip force was correlated to white matter changes in regions associated to the primary motor cortex and the mean grip force applied by MS patients was not changed compared to controls. This implies that the increase in GFV in our cohort of subjects was not caused by central paresis but rather due to deficits in the coordination of force output.

However, we acknowledge that this study has several limitations. The correlation analyses performed between FA changes and GFV do not allow a firm conclusion about causal relationships between the affected brain regions and the motor measures. The findings reported above need to be reproduced in a larger cohort of subjects. The regional distribution of changes in FA observed may still be due to selection bias in the group of patients investigated. One obvious limitation is based on the fact that subjects enrolled in this study still need to be capable of grasping and holding the object used. Nevertheless, it seems likely that the deficits in motor performance observed in the measured GFV are at least partly due to the described white matter pathology. We also acknowledge that the patients in our study were recruited irrespective of a relapsing or chronic progressive form of MS. However, although there is evidence that severity and localization of white matter damage differs across MS subtypes [9], it is known that FA detects white matter changes in all subtypes of MS [8]. Due to the limited sample size of our cohort, reliable subgroup analyses could not be performed in this study. In addition, we report cross-sectional analyses only. While the correlation of GFV changes with the EDSS and with white matter, pathology intuitively suggests that GFV may also be used to measure progression of phenotype; this needs to be investigated in a prospective follow-up study. Future studies should also include the MS functional composite score and the nine-hole-peg-test (NHPT), which may provide more sensitive information about fine motor control than the EDSS [10].

In this context it seems noteworthy that GFV has evolved as an objective measure of motor dysfunction in Huntington’s disease: GFV was increased and correlated to the UHDRS-total motor score in symptomatic patients [15] and premanifest gene carriers [37]. GFV increased in the course of symptomatic Huntington’s disease in a small 3-year single centre study [38] and this finding was confirmed in a blinded analysis of quantitative motor data from about 120 patients and 120 control subjects across 2 years in the multicentre biomarker study TRACK-HD [40]. These observations support the feasibility of applying grip force assessments in the setting of prospective multicentre studies.

We conclude that the results of this study support further exploration of GFV as an objective measure of motor disability in MS. Grasping is functionally relevant. The assessment described can be performed repeatedly in outpatient settings without risks for subjects. The sensors used are pre-calibrated, i.e., easily applicable even in multicentre settings. Thus, GFV may evolve as a valuable and sensitive supplemental outcome measure to assess efficacy and side-effects of novel treatments alongside the EDSS, particularly in proof of concept studies. The validity of grip force analysis to assess motor phenotype in MS should be further elucidated in prospective, blinded multicentre studies.
